# Coronavirus Outbreak in Italy: Physiological Benefits of Home-Based Exercise During Pandemic

**DOI:** 10.3390/jfmk5020031

**Published:** 2020-05-07

**Authors:** Silvia Ravalli, Giuseppe Musumeci

**Affiliations:** 1 Department of Biomedical and Biotechnological Sciences, Human, Histology and Movement Science Section, University of Catania, Via S. Sofia n°87, 95123 Catania, Italy; silviaravalli@gmail.com; 2 Research Center on Motor Activities (CRAM), University of Catania, Via S. Sofia n°97, 95123 Catania, Italy; 3 Department of Biology, Sbarro Institute for Cancer Research and Molecular Medicine, College of Science and Technology, Temple University, Philadelphia, PA 19122, USA

**Keywords:** COVID-19, prevention, physical activity, inactivity, home-based exercise, health

## Abstract

The Coronavirus Disease 2019 (COVID-19) pandemic has forced the hardest-hit populations, like Italians, to radically change their daily habits, starting with social distancing, strict preventive measures, and self-isolation. These precautions also apply to sport-related facilities and activities. The difficulty to practice physical activity during this dramatic moment in time adds to the risks associated with sedentary habits, due to staying all the time at home. Here, the importance and the benefits of maintaining exercise routine, even at home, are emphasized in order to avoid the consequences of inactivity.

## 1. Introduction

Someone once said, “Freedom is the most contagious virus known to man”—what about instead when a virus is the most limiting factor to freedom? Worldwide, we are currently facing major difficulties in managing the spread of SARS-CoV-2, with the aim of containing risks and withstanding the pressure on public health system, while at the same time, safeguarding the rights of individuals and avoiding global market collapse. As Europe’s hardest-hit country, Italy has a particularly strong interest in understanding all facets related to the spread of the virus [1].

Since a vaccine is not yet available, prevention measures to avoid contagion, like social distancing and isolation, are the primary strategies for mitigating the spread of the virus. The decree of the Italian Prime Minister on March 9 2020, was issued in order to impose restrictions over the entire country in an attempt to halt the Coronavirus Disease 2019 (COVID-19) rising across Europe [2]. Museums, theatres, cinemas, among other entertainment venues, have been closed all over the country, following the closure of schools, in order to avoid long-term exposure of high-density groups of people in closed and small areas. These containment precautions also apply to sporting events, competitions, gyms, sports clubs, and swimming centers (with the exclusive permission of training sessions of professional athletes), unless practiced outdoors or behind closed doors, without the presence of the public, and under constant monitoring by medical staff.

Despite social concerns and the need for precautions, the will to lead a daily life as regular as possible is natural. We are all creatures of habit, trapped in the routines of our scheduled lunch breaks or the familiarity of our usual parking spot on the street, with little intention of giving in to change, especially when it comes to physical and mental health. This is something that someone who is used to physical exercise may quite well understand. Although maintaining a regular exercise schedule may be of minor concern in respect to other more important priorities, it can be a way to feel in control of your own body, health, and time. The effect of a wise voluntary confinement at home, in these circumstances, can be detrimental not only psychologically but also physically.

## 2. “Sedentary Death Syndrome”

If, in fact, quarantine experience can lead to psychological outcomes such as depression, post-traumatic stress symptoms, panic, confusion, anger, fear, and substance misuse [3], it can also pave the way to several pathophysiological mechanisms arising from inactivity. Physical inactivity can be defined as the range of situations that, due to impossibility of movement (paralysis) or personal habit (long sitting hours), produces decreased energy expenditure toward basal level [4]. It is important to realize that pathologies or worsening of medical conditions caused by inactivity often show their symptoms and manifestations over the long term, and are usually preclinically silent. It is estimated that physical inactivity is responsible, worldwide, for between 6% and 10% of non-communicable disease, including, among others, Parkinson’s disease, autoimmune diseases, strokes, heart diseases, cancers, diabetes, chronic kidney disease, osteoarthritis, osteoporosis, Alzheimer’s disease, and Parkinson’s disease [5]. Physical inactivity also accelerates the loss of functional abilities with aging, leading to decreases in life expectancy, with impacts potentially as large as renowned risks factors such as smoking and obesity.

**Figure 1:**
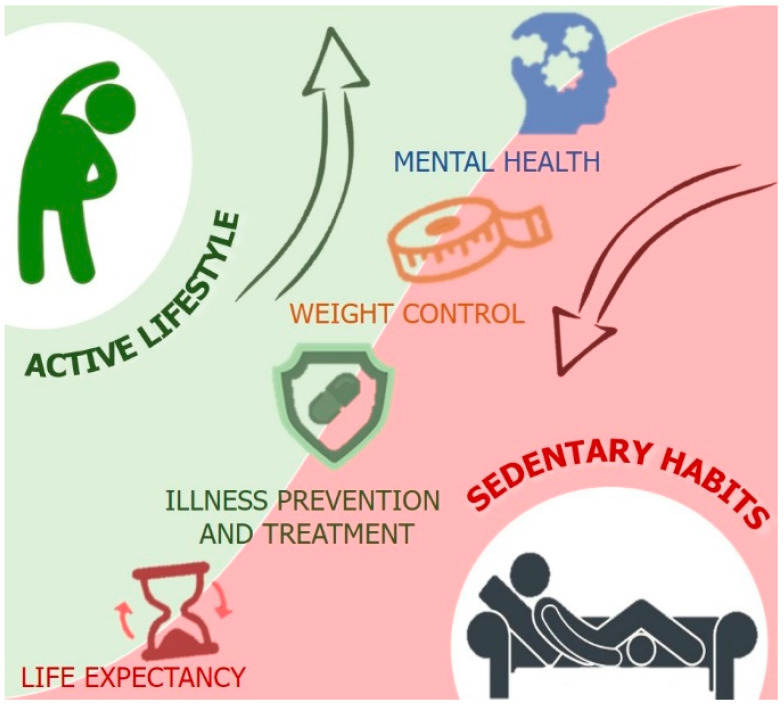
Some aspects of mental and physical well-being that are negatively affected by sedentary habits and positively affected by active lifestyle.

Sedentary habits are difficult to avoid in the modern world due to the progressive mechanization of common working activities, which lets us perform different tasks while sitting in chairs for several hours. Dedicating a few hours a week to exercising is perceived by most as solely a form of free-time activity or as a way to conform to modern beauty paradigms. The term “sedentary death syndrome” was first used to draw attention to this alarming problem, warning about the risks associated with the imbalance between calorie intake and calorie burning, which can lead to hyperinsulinemia and thus adiposity. Overweight, or worse, obesity, are largely associated with an increased risk of cardiovascular problems, osteoporosis, osteoarthritis muscle wasting, and overall physical and mental distress [6,7,8,9]. In this context, physical activity does not only represent an important part of illness prevention but also a treatment for inactivity-associated disorders. Physical activity represents a non-pharmacological approach for promoting general health [10] (Figure 1).

## 3. Exercise under Quarantine

For all of the above reasons, staying at home for self-isolation during this delicate period that we are currently living should not stop us from practicing physical activity, and may instead instill a new and stronger acknowledgment of its importance [11]. Indeed, the closure of gyms and sport facilities can teach us how easy it is to take care of our health anyway, anywhere, and anytime. Opportunities can be seen everywhere; for those who are working from home, for example, the daily commute can be translated in time dedicated to starting the day with stretching and soft strengthening exercises or yoga sessions, which may also be helpful for mental health. Low-, medium-, and high-intensity exercises can also be performed in small rooms, with the help of technologies such as videos and apps that can be freely found on the web or downloaded directly onto a smartphone. For those who are either unable or unwilling to buy it, exercise equipment can be found in daily objects of common use (e.g., bottles of water as weights, chairs as benches, etc.). Stairs can be preferentially chosen over elevators, individuals can opt for long walks to a distant grocery shop instead of traveling by car, and gardening and cleaning can be used as motivational activities to keep oneself active.

For those who are used to exercising, this can be limiting, but running in a park in Italy was allowed for a few weeks after the outbreak, in observance of the recommended minimal distance between people (1 meter) and common-sense precautions to avoid risk of infection, since the virus is transmitted through air droplets and contact with infected people who might not always manifest symptoms. Running in parks was possible until the new decree of the Italian Prime Minister on March 20 2020, which placed further restrictions due to the drastic and exponential growth in the number of infections and deaths as a result of the coronavirus. This decree introduced an absolute ban on walking, ludic, and/or sporting activities in public places, even outdoors. This specific measure was also probably influenced by an unusual social phenomenon of low tolerance towards runners. Those who pursued exercise outside of their homes progressively attracted criticisms from the general population, which saw in their behavior, even beyond rational explanations, a reason for the lack of epidemic improvements, since individuals participating in outdoor activities might serve as possible viral vectors. This is not surprising, as history has taught us that in extreme situations the masses often feel the need to channel their anxiety towards a specific group of individuals. In this context, sportspeople embody the perfect example of “bad citizens”: they assert a right that is not understood or considered essential by many, and they are judged selfish, as they do not fully adhere to the collective sacrifice, whereby individuals are to avoid leaving their homes for reasons other than supplies or work, and because they do not show empathy towards those who cannot leave their homes.

Therefore, home fitness has remained the only solution for being active and performing exercise in Italy in this historical moment of the COVID-19 pandemic. Children can benefit the most from home activities that require the use of the body, since with schools closed their routines can become destabilized, which may cause feelings of stress and alienation, especially when their time may be increasingly spent playing videogames or watching movies. Physical activity is, instead, contraindicated for those who suspect signs of viral infection; general fatigue, fever, sore throat, body aches, and breathing difficulties require medical attention and rest. Respiratory viral infections usually need 3 weeks to recover, and individuals should ease back into performing physical activities only in the absence of symptoms [12]. Just like a drug, physical activity should be, formulated in terms of dosage and administration in order to avoid presenting high-intensity workouts to a body that is not able to adequately respond to and sustain the stresses [13].

## 4. Get Back into a Regular Exercise Routine after Lockdown

After the first half of April 2020, a slight and gradual opening of the restriction measures followed a slowdown in the spread of the virus, allowing physical activity in the immediate vicinity of the home. It is mandatory, however, to stay close to 200 meters from residence, respect the distance of at least one meter from each other person, not create gatherings, and, in some regions, wear a face mask. Since Italy decided to ease coronavirus lockdown beginning May 4 2020, it is expected that people will be able to gradually start practicing exercise more freely.

After the lockdown, physical activity should be gradually phased in so as to avoid excessive tiredness, muscle injury, or health problems, starting from at least 30–45 min every day to a total of 150–300 min per week, as recommended by the American College of Sports Medicine (ACSM), the World Health Organization (WHO), the American Heart Association (AHA), the European network for the promotion of health-enhancing physical activity (HEPA), and by the Italian Ministry of Health’s Physical Activity Guidelines [14,15,16,17]. The intensity of the physical activity must vary according to each individual’s physical conditions and age. Physical reconditioning should provide a training program containing postural, stretching, core stability and balance exercises. Then, exercises should be introduced for increasing muscle tone and a program of resistance activities should be implemented in order to improve aerobic capacity. If the isolation period led to an increase in body weight, it will be paramount to combine the reintroduction of physical activity with a controlled diet—one that is moderately low in calories and rich in vitamins—with appropriate hydration. Furthermore, an evaluation by a sports medicine specialist may be useful for a correct classification of the health conditions and recommendations of physical activities, especially for those with risk factors for chronic diseases or people over 50 years of age. Particular medical attention should be advisable for those who suffered from coronavirus infection.

## 5. How Physical Activity Helps the Immune System

Upper respiratory tract infection (URTI) can be caused by pathogens like coronaviruses, which invade respiratory mucosal tissue and replicate inside the host’s cells. Since viruses, rather than bacteria, are metabolically insufficient, they rely on the host’s cellular machinery to replicate and function. Viruses are highly adaptable and have adopted several ways to avoid detection by the immune system. For this reason, prevention and vaccination are highly important in order to avoid contagion. For most viral illnesses, in fact, the available treatments can only deal with the symptoms, while the immune system response is needed to clear the infection.

A thorough analysis, based upon available evidence, raises the possibility that moderate exercise, which may be able to improve pathological outcomes, could help immune function in resolving viral-induced respiratory infections, like those caused by coronavirus, by inducing the release of stress hormones (catecholamines and glucocorticoids) responsible for reducing excessive local inflammation within the respiratory tract, and by promoting a shifting from a T helper type 1 (Th1) cell population towards a T helper type 2 (Th2) population through the secretion of anti-inflammatory cytokines (e.g., IL-4 and IL-10) in order to prevent excessive prolonged Th1 activity against the pathogen, which can lead to cell damage and necrosis [18].

It is acknowledged that regular and equilibrate exercising improves antibacterial and antiviral immune surveillance, reduces inflammation, and delays immunological aging [19]. The adaptation of immune function depends on the type and duration of exercise and ultimately contributes to the entire wellness status of an individual. The mechanisms behind this enhancement could rely on the stimulated circulation of the innate immune system effectors (immunoglobulins, cytokines (e.g., IL-6), neutrophils, NK cells, cell-mediated immunity by T and B lymphocytes) between lymphoid tissues and the blood circulation, which will lead to better inspection against pathogens, cancer cells, and inflammatory mediators [20]. Epidemiological studies have linked regular training to better immune response to respiratory pathogens such as seasonal influenza, since being physically active, rather that sedentary, helps in managing and limiting the consequences of infection [21]. Exercise as adjuvant intervention seems also to be suggested to improve responses to vaccine prevention strategies, especially in elderly individuals, by enhancing immunoglobulin titer [22].

As for infections, but especially in chronic diseases, the anti-inflammatory effect of training is mostly the result of the release of myokines that stimulate anti-inflammatory signaling pathways, increasing antioxidant defenses, the recruitment of white blood cells, cytokines, and granulocyte-related proteins, and delaying the onset of senescence of immune cells [20].

## 6. Perspectives

It can be concluded that if, on one hand, physical exercise can be beneficial for the defenses of the whole body and may be counted among preventative measures against the consequences of respiratory tract viral infection, that, on the other hand, sedentary habits could be associated with increased risk of predisposition to infections. Hence, maintaining a regular workout routine, carefully in a park setting or safely at home, is a helpful plan to avoid the consequences of inactivity, both at the physical and mental level, and coping with the stress and anxiety derived from the emotional difficulties of this moment. The time spent at home could therefore be translated into an opportunity to build a positive and mindful relationship with the long-term health benefits of exercising.
